# Deep brain stimulation plus best medical therapy versus best medical therapy alone for advanced Parkinson's disease (PD SURG trial): a randomised, open-label trial

**DOI:** 10.1016/S1474-4422(10)70093-4

**Published:** 2010-06

**Authors:** Adrian Williams, Steven Gill, Thelekat Varma, Crispin Jenkinson, Niall Quinn, Rosalind Mitchell, Richard Scott, Natalie Ives, Caroline Rick, Jane Daniels, Smitaa Patel, Keith Wheatley, on behalf of the PD SURG Collaborative Group

**Affiliations:** a Queen Elizabeth Hospital, Birmingham, Birmingham, UK; b Frenchay Hospital, Bristol, UK; c Walton Centre for Neurology and Neurosurgery, Liverpool, UK; d University of Oxford, Oxford, UK; e UCL Institute of Neurology, London, UK; f Russell Cairns Unit, John Radcliffe Hospital, Oxford, UK; g University of Birmingham, Birmingham, UK

## Abstract

**Background:**

Surgical intervention for advanced Parkinson's disease is an option if medical therapy fails to control symptoms adequately. We aimed to assess whether surgery and best medical therapy improved self-reported quality of life more than best medical therapy alone in patients with advanced Parkinson's disease.

**Methods:**

The PD SURG trial is an ongoing randomised, open-label trial. At 13 neurosurgical centres in the UK, between November, 2000, and December, 2006, patients with Parkinson's disease that was not adequately controlled by medical therapy were randomly assigned by use of a computerised minimisation procedure to immediate surgery (lesioning or deep brain stimulation at the discretion of the local clinician) and best medical therapy or to best medical therapy alone. Patients were analysed in the treatment group to which they were randomised, irrespective of whether they received their allocated treatment. The primary endpoint was patient self-reported quality of life on the 39-item Parkinson's disease questionnaire (PDQ-39). Changes between baseline and 1 year were compared by use of *t* tests. This trial is registered with Current Controlled Trials, number ISRCTN34111222.

**Findings:**

366 patients were randomly assigned to receive immediate surgery and best medical therapy (183) or best medical therapy alone (183). All patients who had surgery had deep brain stimulation. At 1 year, the mean improvement in PDQ-39 summary index score compared with baseline was 5·0 points in the surgery group and 0·3 points in the medical therapy group (difference −4·7, 95% CI −7·6 to −1·8; p=0·001); the difference in mean change in PDQ-39 score in the mobility domain between the surgery group and the best medical therapy group was −8·9 (95% CI −13·8 to −4·0; p=0·0004), in the activities of daily living domain was −12·4 (−17·3 to −7·5; p<0·0001), and in the bodily discomfort domain was −7·5 (−12·6 to −2·4; p=0·004). Differences between groups in all other domains of the PDQ-39 were not significant. 36 (19%) patients had serious surgery-related adverse events; there were no suicides but there was one procedure-related death. 20 patients in the surgery group and 13 in the best medical therapy group had serious adverse events related to Parkinson's disease and drug treatment.

**Interpretation:**

At 1 year, surgery and best medical therapy improved patient self-reported quality of life more than best medical therapy alone in patients with advanced Parkinson's disease. These differences are clinically meaningful, but surgery is not without risk and targeting of patients most likely to benefit might be warranted.

**Funding:**

UK Medical Research Council, Parkinson's UK, and UK Department of Health.

## Introduction

Parkinson's disease is caused in part by loss of dopaminergic neurons in the substantia nigra pars compacta; the resultant abnormal neuronal oscillatory and synchronous activity between the subthalamic nucleus, globus pallidus pars interna, and cerebral cortex leads to increasing problems with tremor, rigidity, bradykinesia, and postural disturbances.^1^ Levodopa and other dopaminergic drugs relieve these movement disorders,^2^ but dyskinesia and motor fluctuations develop after a few years.

Most neurosurgery for Parkinson's disease has been done on the thalamus, globus pallidus pars interna, or subthalamic nucleus, using either lesioning or high frequency deep brain stimulation. In recent years, advances in imaging have increased the precision of surgical interventions; this and advances in the understanding of basal ganglia physiology^3^, ^4^, ^5^ have meant that deep brain stimulation of the subthalamic nucleus has been preferred.^6^

In the late 1990s, there was little reliable evidence from randomised trials on the efficacy and safety of surgery.^7^ Thus, we started the PD SURG trial with the aim of comparing the effect of surgery with best medical therapy in patients with advanced Parkinson's disease. This report presents the results at 1 year's follow-up.

## Methods

### Patients

PD SURG is a randomised, open-label trial. Patients with Parkinson's disease for whom current medical therapy was not providing adequate symptomatic control were eligible. Inclusion criteria were diagnosis of Parkinson's disease according to the UK Brain Bank criteria,^8^ age-adjusted score of greater than 5 on the dementia rating scale-II (DRS-II),^9^ and fitness for surgery.

For the **trial protocol** see http://www.pdsurg.bham.ac.uk/investigators/documentation

All patients gave written informed consent before randomisation. The trial was approved by the West Midlands multicentre research ethics committee and local ethics committees at each centre.

### Randomisation and masking

Patients were randomly assigned by a telephone call made to the central trial office. Allocation (1:1) to surgery and best medical therapy (surgery group) or best medical therapy alone (medical therapy group) was done by use of a computerised minimisation procedure with the following categories: age at entry (<60, 60–69, and ≥70 years); years since diagnosis of Parkinson's disease (<5, 5–9, 10–14, and ≥15 years); Hoehn and Yahr stage^10^ in the on state (≤2·0, 2·5, 3·0, and ≥4·0); reason for considering surgery (tremor, dyskinesia, severe off periods, or other reasons); type of surgery (stimulation or lesion) and region to be targeted if allocated to surgery (subthalamic nucleus or globus pallidus pars interna); and drug therapy to be given if allocated to medical therapy (apomorphine or other standard drug treatments for Parkinson's disease). A pair-wise randomisation option was available so that centres could enter two patients together,^11^ with one allocated to surgery and one to medical therapy. Patients and clinicians were unmasked to treatment allocation.

### Procedures

Patients allocated to surgery could receive any standard procedure in use at the time: either stimulation or lesioning of either the subthalamic nucleus or globus pallidus pars interna. Surgery was to be done within 4 weeks of random allocation. The local clinician selected the surgical techniques and postoperative management of stimulator settings for each patient.

Patients in both groups received medical therapy, which could include apomorphine according to local practice, other dopamine agonists, monoamine oxidase type B inhibitors, catechol-*O*-methyltransferase inhibitors, amantadine, or other drugs for treatment of Parkinson's disease symptoms. Levodopa equivalents were calculated on the basis of 100 mg/day of standard levodopa being equivalent to the following doses of other drugs: 133 mg controlled-release levodopa; 1 mg pergolide, pramipexole, cabergoline, or rasagiline; 1·25 mg sublingual selegiline; 2 mg benzhexol; 3·3 mg rotigotine; 5 mg ropinirole; 10 mg bromocriptine, oral selegiline, or apomorphine; and 100 mg amantadine. The total levodopa dose was multiplied by 1·33 for entacapone and by 1·5 for tolcapone.

Apart from the random treatment allocation, all other aspects of the management of patients were at the discretion of the local clinicians. Patients in the medical therapy group could cross over to receive surgery after about 1 year.

The primary endpoint was the patient's self-evaluation of their functional status by use of the 39-item Parkinson's disease questionnaire (PDQ-39).^12^ Secondary endpoints included clinical assessment of functioning (unified Parkinson's disease rating scale [UPDRS]^13^ in both on and off states) and cognitive status (DRS-II).^9^ The UPDRS was assessed in the on state (on medication) and off state (after overnight withdrawal of medication) at study entry, and in the on state (on medication and on stimulation if surgery was done) and off state (after overnight withdrawal of medication but on stimulation if surgery was done) at follow-up. Neuropsychological assessments were also done in a subset of patients and involved a clinical interview and a battery of 16 psychometric tests and questionnaires. Neuropsychological assessment could not be done on all patients because trained examiners were not available in some centres. For centres that did not have trained examiners, a similar method to that used in a previous multicentre randomised controlled trial was adopted:^14^ where possible, psychologists (who were based, trained, and supervised centrally in Oxford) visited the centres to complete assessments as required. We collected data on the type and dose of drug treatments for Parkinson's disease and on the incidence of serious adverse events (defined as any event that resulted in a prolonged stay in hospital or admission to hospital, was thought to be life-threatening, or resulted in death). Data on serious adverse events was collected on serious adverse events forms, annual follow-up forms (completed by the local clinician), resource usage forms (completed by the patients), and partial review of medical notes and information from family doctors. Data for non-serious adverse events was also collected for patients in the surgery group by use of post-operation forms (one immediately after surgery and one 6 months later) and subsequent annual follow-up forms. Patients in both groups were to be assessed at 1, 2, 3, 5, 7, and 9 years after randomisation. Here, we present data from the 1-year follow-up.

### Statistical analysis

PD SURG was designed to detect a ten-point difference (regarded as clinically important) between groups in the PDQ-39 summary index. Assuming a standard deviation of 30 (two-sided p of 0·05 and 90% power), this required random allocation of about 400 patients in total.

An independent data monitoring committee reviewed efficacy and safety data annually. If large differences between the groups were observed, the data monitoring committee could recommend to the independent trial steering committee that enrolment to the trial be stopped early or modified as appropriate.

Patients were analysed in the treatment group to which they were randomised, irrespective of whether they received their allocated treatment, although patients without follow-up at 1 year could not be included in the analysis. For continuous variables, changes from baseline to 1 year were compared between the groups using *t* tests. Missing values in PDQ-39 domain scores were imputed by use of the expectation maximisation algorithm.^15^ Categorical data were analysed using χ^2^ tests or Fisher's exact tests. Subgroup analyses by protocol-specified stratification parameters were done to explore differences in treatment effect across subgroups, using tests of heterogeneity or tests for trend. Analyses were done using SAS version 9.1 (Cary, NC, USA).

This trial is registered with Current Controlled Trials, number ISRCTN34111222.

### Role of the funding source

The study funding sources were not involved in the study design, data collection, data analysis, data interpretation, or the writing of the report, nor were they involved in the decision to submit the paper for publication. The manufacturers of the stimulators used in the trial had no role in the design, data collection, data analysis, data interpretation, writing of the report, or the decision to submit the paper for publication. The views expressed in this Article do not necessarily reflect those of the funding bodies. All authors had full access to the study data, read and approved the final version of the paper, and were responsible for the decision to submit the paper for publication.

## Results

**Figure 1:**
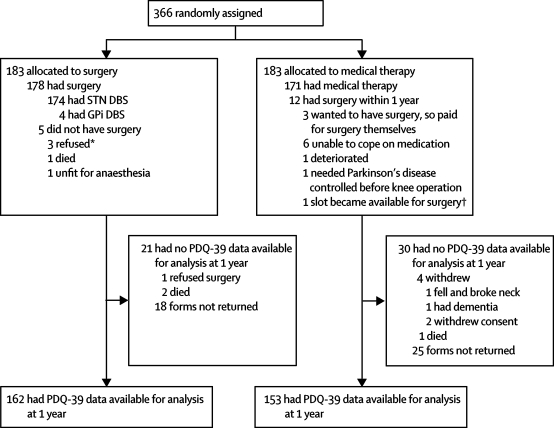
Trial profile PDQ-39=39-item Parkinson's disease questionnaire. STN=subthalamic nucleus. DBS=deep brain stimulation. GPi=globus pallidus pars interna. *1 patient who refused surgery then withdrew from the trial. †As slots for surgery became available around the 1 year timepoint they were used for patients in the medical therapy group. In one case, a slot became available earlier and the treating clinician decided to cross this patient over early (at 10 months).

Between November, 2000, and December, 2006, 366 patients from 13 neurosurgical centres in the UK were randomly assigned to the surgery group or to the best medical therapy group (183 per group, figure 1). Baseline characteristics were similar between groups (Table 1, Table 2, Table 3). 348 of 366 patients were aged less than 70 years (mean age 59 years) and 341 patients had had Parkinson's disease for at least 5 years (mean duration 11·4 years). Dyskinesia (n=267) and severe off periods (n=280) were the most common reasons for considering surgery (table 1). In addition to levodopa, 357 of 366 patients had received previous therapy with a dopamine agonist, 197 with a monoamine oxidase type B inhibitor, 214 with a catechol-*O*-methyltransferase inhibitor, and 145 with apomorphine (90 were still on apomorphine at random allocation).

**Table 1:** Demographics and baseline characteristics

		**Surgery (n=183)**	**Medical therapy (n=183)**
**Demographics**			
Age (years)		59 (37–79)	59 (36–75)
Men		125 (68%)	135 (74%)
**Baseline characteristics**			
Duration of Parkinson's disease (years)		11·5 (2·0–32·2)	11·2 (1·0–30·0)
Hoehn and Yahr stage (on state)			
	≤2	66 (36%)	68 (37%)
	2·5	58 (32%)	55 (30%)
	3	54 (30%)	54 (30%)
	4	5 (3%)	6 (3%)
	5	0 (0%)	0 (0%)
Hoehn and Yahr stage (off state)*			
	≤2	12 (7%)	11 (6%)
	2·5	19 (11%)	29 (16%)
	3	65 (38%)	59 (34%)
	4	54 (32%)	55 (31%)
	5	19 (11%)	22 (13%)
Previous treatment†			
	Dopamine agonist	179 (98%)	178 (97%)
	Monoamine oxidase type B inhibitor	99 (54%)	98 (54%)
	Catechol-*O*-methyltransferase inhibitor	103 (56%)	111 (61%)
	Apomorphine	72 (39%)	73 (40%)
	On apomorphine at study entry	45 (25%)	45 (25%)
Reason for considering surgery†			
	Tremor	73 (40%)	73 (40%)
	Dyskinesia	134 (73%)	133 (73%)
	Severe off periods	141 (77%)	139 (76%)
	Other‡	16 (9%)	17 (9%)
PDQ-39 summary index		37·5 (14·6)	38·7 (13·7)
UPDRS part III: motor (on)		18·9 (11·4)	20·1 (11·4)
UPDRS part III: motor (off)		47·6 (14·0)	48·6 (14·3)

Data are mean (range), number (%), or mean (SD). UPDRS=unified Parkinson's disease rating scale. PDQ=Parkinson's disease questionnaire.

* Based on baseline UPDRS part IV: complications of therapy, where patients are asked what their worst Hoehn and Yahr stage had been in the past week. Data were available for 169 of 183 patients in the surgery group and 176 of 183 patients in the medical therapy group.

† Not mutually exclusive: more than one previous treatment and reason for considering surgery could apply.

‡ Including motor complications (fluctuations or dystonia, n=12), rigidity (n=7), bradykinesia (n=5), gait problems (n=4), intolerance to drugs (n=3), goose stepping (n=1), and pain (n=1). Percentage values do not add up to 100 in some cases because of rounding.

**Table 2:** Primary outcome (PDQ-39)

	**Baseline**		**1 year**				**Change between baseline and 1 year**			
	Surgery (n=178)	Medical therapy (n=178)	Surgery (n=162)	Medical therapy (n=153)	Difference in means at 1 year (95% CI)	p	Surgery (n=160)	Medical therapy (n=150)	Difference in the mean change (95% CI)	p
Summary index	37·5 (14·6)	38·7 (13·7)	32·5 (15·8)	38·1 (13·5)	−5·6 (−8·9 to −2·4)	0·0008	−5·0 (14·1)	−0·3 (11·1)	−4·7 (−7·6 to −1·8)	0·001
Mobility	56·3 (22·5)	60·1 (22·4)	48·1 (25·2)	60·2 (23·6)	−12·0 (−17·5 to −6·6)	<0·0001	−8·2 (24·8)	0·7 (18·9)	−8·9 (−13·8 to −4·0)	0·0004
Activities of daily living	49·6 (21·4)	51·4 (20·4)	37·0 (21·6)	51·0 (21·1)	−14·0 (−18·7 to −9·3)	<0·0001	−12·3 (23·6)	0·1 (20·3)	−12·4 (−17·3 to −7·5)	<0·0001
Emotional wellbeing	31·3 (19·7)	31·0 (19·5)	27·9 (21·1)	28·6 (18·8)	−0·7 (−5·1 to 3·8)	0·77	−3·3 (20·9)	−1·2 (16·2)	−2·1 (−6·3 to 2·1)	0·33
Stigma	33·2 (25·2)	37·2 (26·0)	25·5 (24·2)	35·0 (24·6)	−9·5 (−14·9 to −4·1)	0·0006	−8·1 (24·4)	−3·0 (21·9)	−5·2 (−10·4 to 0·03)	0·05
Social support	18·8 (19·0)	16·6 (20·9)	19·2 (20·5)	16·8 (19·3)	2·4 (−2·1 to 6·8)	0·30	0·6 (18·4)	0·5 (19·3)	0·1 (−4·1 to 4·4)	0·95
Cognition	30·4 (19·7)	29·6 (20·5)	28·6 (21·3)	30·0 (19·4)	−1·4 (−5·9 to 3·1)	0·54	−1·7 (19·7)	1·4 (19·2)	−3·0 (−7·4 to 1·3)	0·17
Communication	31·9 (23·1)	31·7 (23·1)	34·3 (23·0)	33·0 (21·0)	1·3 (−3·6 to 6·2)	0·61	2·9 (22·3)	1·6 (18·0)	1·4 (−3·2 to 5·9)	0·55
Bodily discomfort	48·9 (23·1)	52·1 (23·8)	39·2 (23·7)	50·0 (23·0)	−10·9 (−16·1 to −5·7)	<0·0001	−9·8 (23·1)	−2·4 (22·6)	−7·5 (−12·6 to −2·4)	0·004

Data are mean (SD). The PDQ-39 range is 0–100; the higher the score, the worse the self-reported quality of life; negative change=improvement. Ten baseline forms and 51 1-year forms were not returned. Five patients returned 1-year PDQ-39 forms, but did not return baseline PDQ-39 forms, so we were unable to calculate a change from baseline for these patients. Missing values in PDQ-39 domain scores were imputed using the expectation maximisation algorithm. PDQ-39=39-item Parkinson's disease questionnaire.

**Table 3:** UPDRS and DRS-II scores

	**Baseline**				**1 year**						**Change between baseline and 1 year**					
	**Surgery**		**Medical therapy**		**Surgery**		**Medical therapy**		**Difference in means at 1 year (95% CI)**	**p**	**Surgery**		**Medical therapy**		**Difference in the mean change (95% CI)**	**p**
	n	Mean (SD)	n	Mean (SD)	n	Mean (SD)	n	Mean (SD)			n	Mean (SD)	n	Mean (SD)		
UPDRS*																
Part I: mental	176	2·3 (1·7)	181	2·2 (1·6)	154	2·3 (1·8)	151	2·5 (1·7)	−0·2 (−0·6 to 0·2)	0·29	148	−0·01 (1·8)	149	0·3 (1·6)	−0·3 (−0·7 to 0·1)	0·15
Part II: activities of daily living (on)	170	9·9 (6·5)	172	10·3 (6·0)	150	9·9 (5·9)	146	10·9 (6·5)	−1·0 (−2·4 to 0·4)	0·16	142	0·3 (6·4)	138	1·0 (5·1)	−0·6 (−2·0 to 0·7)	0·36
Part II: activities of daily living (off)	162	23·8 (7·2)	167	24·7 (7·3)	130	17·6 (8·1)	139	23·9 (7·6)	−6·3 (−8·2 to −4·4)	<0·0001	123	−6·9 (7·2)	129	−0·5 (5·9)	−6·4 (−8·0 to −4·7)	<0·0001
Part III: motor (on)	165	18·9 (11·4)	164	20·1 (11·4)	146	16·0 (8·8)	136	20·4 (10·8)	−4·5 (−6·8 to −2·2)	0·0001	135	−3·3 (9·4)	124	0·8 (9·1)	−4·0 (−6·3 to −1·8)	0·0006
Part III: motor (off)	161	47·6 (14·0)	162	48·6 (14·3)	126	30·6 (15·2)	135	47·3 (15·4)	−16·6 (−20·4 to −12·9)	<0·0001	119	−17·2 (13·1)	123	−0·4 (13·3)	−16·8 (−20·1 to −13·4)	<0·0001
Part IV: complications of therapy	145	9·0 (3·4)	145	9·1 (3·4)	119	4·5 (3·1)	118	9·0 (3·7)	−4·6 (−5·4 to −3·7)	<0·0001	106	−4·4 (3·8)	112	−0·2 (2·8)	−4·2 (−5·1 to −3·3)	<0·0001
Total (I–III) score (on)	161	30·9 (16·6)	159	32·6 (16·3)	141	28·1 (13·8)	132	34·0 (15·6)	−5·9 (−9·5 to −2·4)	0·001	128	−2·8 (14·2)	116	2·5 (11·5)	−5·4 (−8·6 to −2·1)	0·002
Total (I–III) score (off)	155	73·1 (19·7)	158	75·7 (20·0)	121	50·9 (21·3)	127	73·3 (21·7)	−22·4 (−27·8 to −17·1)	<0·0001	114	−23·7 (17·5)	113	−0·5 (18·0)	−23·2 (−27·9 to −18·6)	<0·0001
Total (I–IV) score (on)	136	39·6 (17·8)	129	39·5 (15·4)	111	32·7 (14·6)	107	41·6 (16·3)	−8·9 (−13·0 to −4·7)	<0·0001	95	−6·6 (15·3)	91	1·6 (12·2)	−8·3 (−12·3 to −4·3)	<0·0001
Total (I–IV) score (off)	137	81·5 (21·4)	132	83·6 (21·0)	100	55·6 (22·4)	105	81·9 (24·3)	−26·3 (−32·8 to −19·9)	<0·0001	89	−27·4 (18·9)	91	−0·9 (20·1)	−26·6 (−32·3 to −20·9)	<0·0001
**Cognition**†																
DRS-II	159	10·7 (2·8)	156	10·4 (2·9)	126	10·6 (3·2)	144	10·1 (3·0)	0·5 (−0·3 to 1·2)	0·20	121	−0·4 (3·5)	133	−0·4 (2·9)	0·05 (−0·7 to 0·8)	0·90

UPDRS=unified Parkinson's disease rating scale. DRS-II=dementia rating scale II.

* 177 patients in the surgery group and 181 in the medical therapy group were assessed at baseline, and 155 in the surgery group and 152 in the medical therapy group were assessed at 1 year. Data on 150 patients in each group were included in assessments of mean changes between baseline and 1 year. The numbers analysed for each part of the UPDRS are different because of missing data. There are no imputation methods for the UPDRS. UPDRS score ranges: mental 0–16; activities of daily living 0–52; motor 0–108; complications 0–23; total (parts I–III) 0–176; total (parts I–IV) 0–199 (high scores=worse clinical assessment of the patient's Parkinson's disease). UPDRS negative change=improvement.

† When the trial started patients were allowed to complete either the mini-mental state examination or DRS-II; therefore data on the DRS-II were not available for all patients. DRS-II range: 0–18 (high score=better cognitive function). DRS-II negative change=deterioration.

Five patients in the surgery group did not have surgery: three refused surgery, one was unfit for anaesthesia, and one died before surgery (figure 1). 81 of 178 patients had surgery within 4 weeks of random allocation, 66 within 4–8 weeks, 22 within 8–16 weeks, and nine more than 16 weeks after random allocation. All 178 patients in the surgery group who received surgery had deep brain stimulation and in 174 the subthalamic nucleus was the surgical target. 176 of 178 procedures were bilateral; there was one staged procedure with electrodes implanted 10 months apart.

In the medical therapy group, 12 patients had surgery between baseline and 1 year (figure 1), of whom three received surgery between 10 months and 12 months after random allocation but completed 1-year assessments before surgery. 118 patients randomly allocated to medical therapy had surgery at or after 1 year.

**Figure 2:**
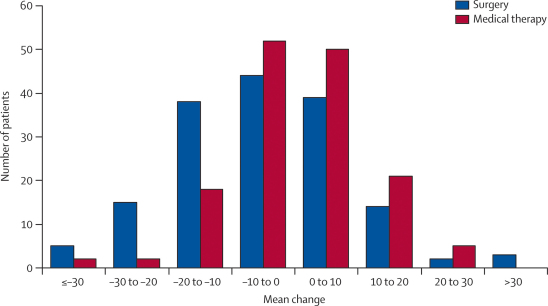
Mean change in 39-item Parkinson's disease questionnaire summary index score between baseline and 1 year Negative scores=improvement.

The mean change between baseline and 1 year on the PDQ-39 summary index was −5·0 points in the surgery group and −0·3 points in the medical therapy group (difference −4·7 points, 95% CI −7·6 to −1·8, p=0·001; table 2). The mean change in PDQ-39 summary index between baseline and 1 year ranged from less than −30 to more than 30 (figure 2). The difference in mean change in score between baseline and 1 year was −8·9 for the PDQ-39 domain of mobility (95% CI −13·8 to −4·0; p=0·0004), −12·4 for activities of daily living (−17·3 to −7·5; p<0·0001), −7·5 for bodily discomfort (−12·6 to −2·4; p=0·004), and −5·2 for stigma (−10·4 to 0·03; p=0·05).

**Figure 3:**
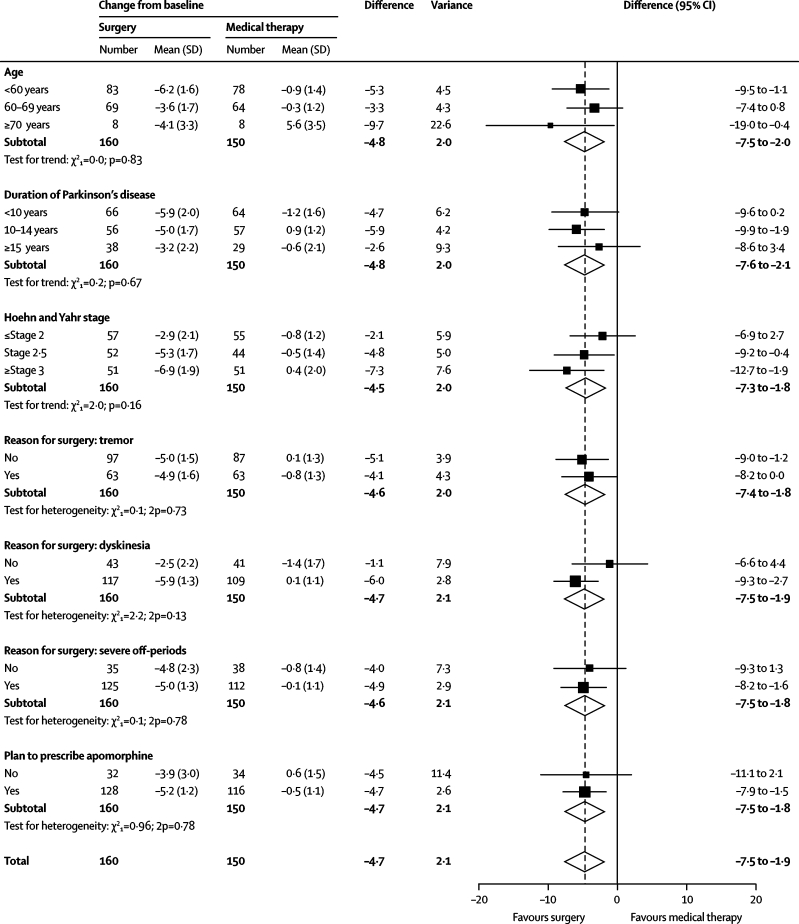
Subgroup analyses of 39-item Parkinson's disease questionnaire summary index score

For the PDQ-39 summary index score, there was no evidence that the size of the treatment effect in favour of surgery varied with age, duration of Parkinson's disease, Hoehn and Yahr stage, reasons for considering surgery, or whether apomorphine treatment was planned (figure 3). Full details of the subgroup analyses will be reported elsewhere.

The mean UPDRS (on) total (parts I–IV) score decreased between baseline and 1 year by 6·6 points in the surgery group (ie, improvement) and increased by 1·6 points in the medical therapy group (difference −8·3 points, 95% CI −12·3 to −4·3; p<0·0001; table 3). The difference in the mean change between baseline and 1 year between the groups was −4·0 (95% CI −6·3 to −1·8; p=0·0006) for the motor (on) subsection of the UPDRS and −4·2 (−5·1 to −3·3; p<0·0001) for the complications of therapy subsection. The mean UPDRS (off) total (parts I–IV) score decreased between baseline and 1 year by 27·4 points in the surgery group and by 0·9 points in the medical therapy group (difference −26·6 points, 95% CI −32·3 to −20·9; p<0·0001). We investigated the answers to UPDRS questions relating specifically to dyskinesia and off periods because these were the two main reasons that patients were considered for surgery. At 1 year, 75 patients in the surgery group and 21 in the medical therapy group reported no waking day dyskinesia (p<0·0001) and 45 in the surgery group and five in the medical therapy group reported no off time (p<0·0001; table 4).

**Table 4:** Dyskinesia and off periods

	**Baseline**			**1 year**		
	Surgery	Medical therapy	p*	Surgery	Medical therapy	p*
**Dyskinesia**						
n	177	180	0·93	155	151	<0·0001
None	19 (11%)	27 (15%)	..	75 (48%)	21 (14%)	..
1–25%	71 (40%)	63 (35%)	..	65 (42%)	56 (37%)	..
26–50%	55 (31%)	55 (31%)	..	10 (6%)	44 (29%)	..
51–75%	26 (15%)	27 (15%)	..	4 (3%)	27 (18%)	..
76–100%	6 (3%)	8 (4%)	..	1 (1%)	3 (2%)	..
**Off time**						
n	175	180	0·40	154	152	<0·0001
None	6 (3%)	2 (1%)	..	45 (29%)	5 (3%)	..
1–25%	66 (38%)	68 (38%)	..	83 (54%)	64 (42%)	..
26–50%	85 (49%)	88 (49%)	..	23 (15%)	63 (41%)	..
51–75%	16 (9%)	20 (11%)	..	3 (2%)	17 (11%)	..
76–100%	2 (1%)	2 (1%)	..	0 (0%)	3 (2%)	..

Based on UPDRS part IV: complications of therapy (Q32: what proportion of the waking day is dyskinesia present? Q39: what proportion of the waking day is the patient off on average?).

* χ^2^ test for the difference between the surgery group and the medical therapy group across all categories.

The DRS-II score decreased by 0·4 points (ie, deterioration) between baseline and 1 year in both groups (difference 0·05, 95% CI −0·7 to 0·8, p=0·90; table 3). 39 measures were compared in the neuropsychological assessments on up to 163 patients. The Delis-Kaplan executive function system phonemic mean score decreased by 6·5 points (SD 9·4) between baseline and 1 year in the surgery group and decreased by 0·6 points (8·7) in the medical therapy group (difference −5·9, 95% CI −8·9 to −2·9; p=0·0002). The mean change in score on the D-KEFS categorical verbal fluency between baseline and 1 year was −4·5 points (SD 7·8) in the surgery group and −0·2 (7·7) in the medical therapy group (difference −4·4, −6·9 to −1·8; p=0·001). On the Wechsler abbreviated scale of intelligence vocabulary, the mean change from baseline to 1 year was −1·5 (4·8) in the surgery group and 0·6 (4·9) in the medical therapy group (difference −2·1, −3·7 to −0·5; p=0·01).

At 1 year, patients in the surgery group were on a mean levodopa equivalent dose of 894 mg/day (SD 568) and those in the medical therapy group were on 1347 mg/day (585, p<0·0001). This difference of 453 mg/day (95% CI 328 to 580) at 1 year represents a 34% reduction in mean drug dose in the surgery group compared with the medical therapy group.

At baseline, 45 patients in each group were on apomorphine. By 1 year, this had decreased to 13 in the surgery group (ten were on apomorphine at baseline and three started on apomorphine after random allocation) and had increased to 63 in the medical therapy group (34 were on apomorphine at baseline and 29 started on apomorphine after randomisation). Of the patients on apomorphine at 1 year, 54 patients were on continuous drug infusions (six in the surgery group and 48 in the medical therapy group) and 22 were on intermittent dosing (seven in the surgery group and 15 in the medical therapy group).

36 of 178 patients in the surgery group had 43 surgery-related serious adverse events. There were no serious adverse events in the 12 patients in the medical therapy group who received surgery in the first year. The most common surgery-related serious adverse events were infections (n=16; table 5).

**Table 5:** Serious adverse events in the first year

			**Surgery (n=183)**	**Medical therapy (n=183)**
Surgery-related			43 events in 36 patients	0 events in 12 patients*
	Haemorrhage		4 (including 1 death)†	0
	Infection		16	0
	DBS-specific adverse events		13 events in 12 patients	0
		Postoperative confusion	5	0
		Neck pain	2	0
		Seizures	2	0
		Deteriorating control of Parkinson's disease because battery was switched off	1	0
		Psychosis	1	0
		Unresponsive on operating table (possibly because of levodopa withdrawal)	1	0
		Visual neglect from oedema	1	0
	General surgery problems		10 events in 9 patients	0 events
		Urinary retention	4	0
		Pulmonary embolism	2	0
		Anxiety attack	1	0
		Difficulty removing catheter	1	0
		Postoperative hypotension	1	0
		Pyrexia	1	0
Parkinson's-disease related and drug-related			25 events in 20 patients	14 events in 13 patients
	Falls		3	7
	Constipation		4 in 3 patients	2
	Worsening of Parkinson's disease symptoms or uncontrolled Parkinson's disease symptoms		12 in 11 patients	2
	Psychiatric problems		4	1
		Neuropsychiatric disturbances (including hallucinations or paranoia)	3	0
		Breakdown	0	1
		Suicide attempt	1	0
	Parkinson's disease drug-related		2	2
Other			26 events‡ in 19 patients	14 events§ in 13 patients
Deaths			2 (haemorrhage and pneumonia)	1 (stroke)
Total			96 events in 65 patients	29 events in 26 patients

Serious adverse events were any event that prolonged a patient's stay in hospital, resulted in the patient being admitted to hospital, was considered to be life-threatening, or resulted in death. DBS=deep brain stimulation.

* 12 patients randomly assigned to medical therapy received surgery between baseline and 1 year.

† One patient had a haemorrhage 5 months after surgery and this was probably not treatment related.

‡ Five urinary problems; five leg swelling or knee swelling, or both; three pain; two chest pain or angina; two chest infection; one collapse; one deep vein thrombosis (more than 8 months after surgery); one pulmonary embolism (more than 8 months after surgery); one polymyalgia rheumatica; one vertigo; one renal colic; one fainting episode; one lacerated wound to forearm; and one head injury.

§ Four chest pain or angina; two urinary problems; two pain; one abscess on chest wall leading to infection; one deep vein thrombosis; one cauda equina syndrome; one fainting episode; one confusion; and one chest problems.

There were 39 Parkinson's disease-related and drug-related serious adverse events reported in 33 patients (25 events in 20 patients in the surgery group and 14 in 13 patients in the medical therapy group), the most common of which were worsening of Parkinson's disease symptoms or uncontrolled Parkinson's disease symptoms (12 events in 11 patients in the surgery group and two in the medical therapy group; table 5). There was one unsuccessful postoperative suicide attempt in a patient in the surgery group; however, this patient had previously attempted suicide before trial entry. Three patients died during the first year: one from haemorrhage during surgery, one in the surgery group from pneumonia 3 weeks after study entry before surgery was done, and one from stroke in the medical therapy group 10 months after entry.

## Discussion

PD SURG was designed with quality of life as the primary endpoint to examine the efficacy of deep brain stimulation versus medical therapy on the daily lives of people with Parkinson's disease and thus provide practical information to inform future use. PD SURG included a representative sample of patients likely to be offered surgery at neuroscience centres in the UK, where apomorphine is readily available, thus enabling comparison of surgery with best medical therapy and providing evidence on the benefits of surgery in a real-world setting. The follow-up reported here was longer than in two other large trials of deep brain stimulation versus medical therapy,^16^, ^17^ and thus gives statistically more reliable results and provides evidence on the longer term benefits of surgery (with less likelihood of a so-called honeymoon effect^18^ in the period just after surgery). Although one trial has investigated the effects of surgery for Parkinson's disease to 18 months, only 20 patients were included and thus the trial was underpowered; also, patients with earlier stage disease were recruited.^19^

From a purely scientific perspective, a long-term trial of surgery versus medical therapy would have been ideal; however, a realistic design acceptable to both patients and clinicians was necessary, and so surgery was permitted after 1 year in the medical therapy group. There were clear advantages for surgery compared with medical therapy alone at 1 year, both in patient-assessed quality of life and on clinical assessment. These benefits are likely to be meaningful to patients, as measured by use of the PDQ-39,^20^ and were found in domains of the PDQ-39 that surgery would be expected to affect (eg, mobility and activities of daily living), but not in others (eg, social support, cognition, and communication). These findings were mirrored by clinically meaningful differences on the UPDRS,^21^ including the patient-rated UPDRS part IV, which showed substantial benefits of surgery in the time and severity of dyskinesia and off periods—the most common reasons for patients to be considered for surgery. Greater benefits for the surgery group than the medical therapy group were seen for off-medication UPDRS assessments. However, this represents an artificial situation, created by a temporary withdrawal of medication and does not indicate an absence of drug because the washout period was not long enough. When considering the real-life on-medication assessment, the magnitude of the benefit seen in our trial is smaller than perhaps anticipated from the numerous small uncontrolled series that have suggested large effects of surgery.^22^, ^23^

**Figure 4:**
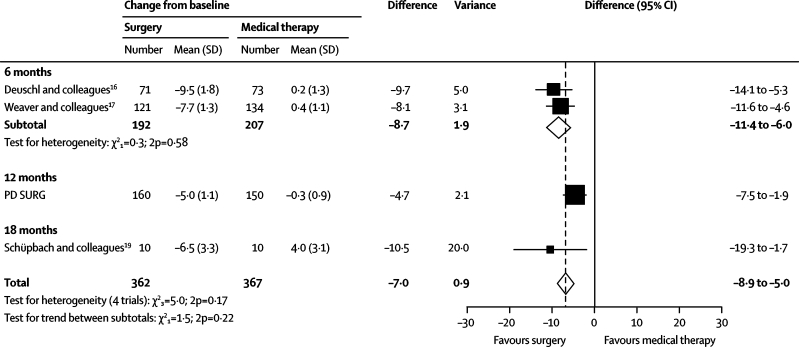
Meta-analysis of 39-item Parkinson's disease questionnaire summary index score in trials of deep brain stimulation versus medical therapy

PD SURG, along with other reported randomised trials,^16^, ^17^, ^19^ shows benefits for surgery over best medical treatment in patients with advanced Parkinson's disease, even when apomorphine is available, while also confirming that there are risks associated with surgery. A meta-analysis of PDQ-39 summary index scores showed that the results of the trials are generally consistent with each other (test for heterogeneity, p=0·2; figure 4), although there is evidence of heterogeneity of treatment effect between the trials with 6 months of follow-up and PD SURG with 12 months of follow-up (test for interaction, p=0·04).

The PD SURG results at 1 year show smaller differences between the groups in the PDQ-39 summary index (4·7 points) than was seen in the two trials that reported results after 6 months (8·7 points).^16^, ^17^ Although from a statistical perspective this difference is not substantial and might be a chance effect, it is worth considering potential alternative explanations based on differences in the trial designs. First, it is possible that there is a large immediate effect of surgery, whether real or in part related to an early so-called honeymoon effect,^18^ which gradually decreases over time. If the benefits of surgery are relatively transient, this would call into question the long-term value of surgery. Second, there might have been better drug treatment of Parkinson's disease in the medical group of PD SURG than in the other two trials, because of the use of apomorphine in over one-third of patients in this group. Apomorphine is an effective drug in advanced Parkinson's disease^2^ and can be given by continuous infusion to enable a more constant dose to be delivered to the patient, thereby smoothing out on–off periods and fluctuations. However, apomorphine is expensive, and thus in the UK tends to be used only when other drugs have failed to control the symptoms of Parkinson's disease adequately; that is, it might be used in the same situations as surgery for patients with advanced Parkinson's disease. Hence, a comparison of the effects of surgery plus medical therapy versus medical therapy, in a population of patients whose treatment could have included apomorphine (as in PD SURG), provides better evidence on the relative benefits of surgery than a comparison with medical therapy not including apomorphine. However, apomorphine is less widely used outside the UK, and was not reported as being widely used in the other trials.^16^, ^17^ Administration of apomorphine is more complicated than for other Parkinson's disease drugs, requiring infusion and monitoring. Nevertheless, because of its efficacy, apomorphine use might become more common, and thus the results of PD SURG could have wider relevance in future. Optimisation of medical therapy might lead to a smaller comparative advantage for surgery. Nevertheless, surgery is still a valid treatment because patients would need to have only a one-off procedure (albeit with need for stimulator adjustment and replacement) rather than regular administration of an expensive drug. Whether technical aspects of the procedure, such as electrode location within the target site, are factors that could be improved are also important to consider.^24^, ^25^

Substantially more patients in the surgery group had serious adverse events than did patients in the medical therapy group, confirming that deep brain stimulation surgery for Parkinson's disease is not without risks.^26^, ^27^ Reporting of all serious adverse events, whether surgery related, disease related, or drug related, was mandatory in both the surgical and medical groups. Because a 6-month postoperation form that included serious adverse events was completed only in the surgical group, there could have been differential reporting of serious adverse events unrelated to surgery, despite efforts—through a case-note review—to collect these data in both groups. However, similar surgery-related serious adverse events were seen in the two other trials.^16^, ^17^ Furthermore, recently raised concerns about the suicide rate^27^, ^28^, ^29^ after surgery were not confirmed in our study (only one patient attempted suicide after surgery), and only one patient died as a result of the procedure. A limitation of the study is that adverse events that were not serious enough to cause or prolong a patient's stay in hospital were not routinely recorded. However, adverse events are difficult to record accurately (eg, there were three times more adverse events in one of two comparable trials than in the other)^16^, ^17^ and their combined effects should be reflected in the participants' perception of their quality of life.

Preliminary analysis of the neuropsychological outcomes, to be reported in detail elsewhere, did not suggest any major adverse effect of surgery other than on verbal fluency and vocabulary. The changes in group means for neuropsychological outcomes represent small decreases in individual scores that are not usually associated with clinically meaningful effects on any activities of daily living.^30^ Subclinical decreases in verbal fluency after deep brain stimulation or lesional surgery for Parkinson's disease have been reported,^31^, ^32^, ^33^ and are understood to be caused by disruption to projections from the basal ganglia to the prefrontal cortex, which are involved in language and executive skills. For example, after deep brain stimulation of the subthalamic nucleus, substantial associations have been reported between activation of areas including the dorsolateral prefrontal cortex and Broca's area, as measured with fluorine-18-labelled-fluorodeoxyglucose-PET, and performance on verbal fluency tasks.^33^ These changes are not associated with patient age, disease duration, or dose of dopaminomimetic drugs after surgery;^34^ however, a frequency-dependent reciprocal modulation of verbal fluency and motor functions in deep brain stimulation of the subthalamic nucleus has been reported.^34^

Discussions with potential candidates for surgery should include the potential risks and benefits of surgery. Deep brain stimulation is a costly procedure and therefore health economic issues need to be taken into account. However, the amount of drug therapy required in the surgery group was about one third lower than the amount required by those in the medical therapy group. Thus, the cost of surgery will be partly offset by the reduction in the amount of drug therapy required by patients who have had surgery. In particular, if apomorphine or continuous intestinal infusions of levodopa, with high recurrent costs, are the alternative drug treatment options, the cost-effectiveness equation might favour surgery (a full economic analysis of PD SURG will be reported elsewhere). Thus, it is important to identify patients who are or are not likely to benefit from surgery when the risks and costs are taken into account. Subgroup analyses are unreliable, with a high likelihood of chance effects being observed.^35^ The protocol-specified subgroup analyses did not provide clear evidence that the benefit of surgery differed in different types of patient, although, given the insensitivity of tests for interaction,^36^ the possible greater benefit in patients with more advanced disease, as measured by Hoehn and Yahr stage (p=0·2), is worthy of further investigation.

Some limitations in the design of PD SURG should be acknowledged, especially in relation to a potential placebo effect. Ideally, patients and assessors would have been masked to treatment allocation. However, sham surgery on patients in the medical therapy group (ie, to insert electrodes and stimulators but not switch them on) would not have been practical (eg, increased theatre time and cost). Furthermore, attempts at masking are likely to be ineffective because, in many cases, patients will be able to tell if their stimulator is switched on. Thus, patients' perception of their quality of life could have been influenced by their knowledge of the treatment they received. Use of independent masked assessors was beyond the resources available for this trial and, because the UPDRS was a secondary endpoint, was not considered essential. The use of the insensitive DRS-II as a measure of cognition was also a potential drawback, but this was deemed to be adequate to provide an overall assessment of the whole trial population, with a more detailed neuropsychological evaluation being done in a subset of patients. The absence of a standard definition of the on state in the protocol might also have been a limitation of the study, although all centres had experienced neurological teams familiar with doing the UPDRS and the comparative nature of the trial meant that any cross-centre differences would apply to both groups and would not introduce bias.

Follow-up of PD SURG will continue for several years and future papers will report on the longer term outcome of immediate surgery versus deferred surgery; subgroup and prognostic factor analyses; neuropsychological effects of surgery; the effect of surgery on carers; further details on the procedure and longer term outcomes; and health economic evaluation. Surgery is likely to remain an important treatment option for patients with Parkinson's disease, especially if the way in which deep brain stimulation exerts its therapeutic effects is better understood, if its use can be optimised by better electrode placement and settings, and if patients who would have the greatest benefit can be better identified.
